# Localized Expression of Olfactory Receptor Genes in the Olfactory Organ of Common Minke Whales

**DOI:** 10.3390/ijms25073855

**Published:** 2024-03-29

**Authors:** Ayumi Hirose, Gen Nakamura, Masato Nikaido, Yoshihiro Fujise, Hidehiro Kato, Takushi Kishida

**Affiliations:** 1 School of Life Science and Technology, Tokyo Institute of Technology, Tokyo 152-8550, Japan; mnikaido@bio.titech.ac.jp; 2 Department of Ocean Sciences, Tokyo University of Marine Science and Technology, Tokyo 108-8477, Japan; 3 The Institute of Cetacean Research, Tokyo 104-0055, Japan; 4 Museum of Natural and Environmental History, Shizuoka 422-8017, Japan; kishida.takushi@nihon-u.ac.jp; 5 College of Bioresource Sciences, Nihon University, Fujisawa 252-0880, Japan

**Keywords:** epithelium, histology, Mysticeti, nasal complex, olfaction, RNA-seq, secondary-aquatic

## Abstract

Baleen whales (Mysticeti) possess the necessary anatomical structures and genetic elements for olfaction. Nevertheless, the *olfactory receptor* gene (*OR*) repertoire has undergone substantial degeneration in the cetacean lineage following the divergence of the Artiodactyla and Cetacea. The functionality of highly degenerated mysticete *OR*s within their olfactory epithelium remains unknown. In this study, we extracted total RNA from the nasal mucosae of common minke whales (*Balaenoptera acutorostrata*) to investigate *OR*s’ localized expression. All three sections of the mucosae examined in the nasal chamber displayed comparable histological structure. However, the posterior portion of the frontoturbinal region exhibited notably high *OR* expression. Neither the olfactory bulb nor the external skin exhibited the expression of these genes. Although this species possesses four intact non-class-2 *OR*s, all the *OR*s expressed in the nasal mucosae belong to class-2, implying the loss of aversion to specific odorants. These anatomical and genomic analyses suggest that *OR*s are still responsible for olfaction within the nasal region of baleen whales, enabling them to detect desirable scents such as prey and potential mating partners.

## 1. Introduction

Olfaction, the sense of smell, is one of the sensory modalities encompassing biologically important behaviors such as foraging, predator avoidance, mother–calf relationships, mating, and territorial display [1,2,3]. The sense of smell arises when olfactory receptor proteins within the nasal cavity capture volatile chemical substances. These proteins are localized on the membranes of olfactory cells. Stimulation is subsequently transmitted from the receptor protein through the cribriform plate into the main olfactory bulb by olfactory nerves, extending from the base of olfactory cells. Olfactory receptor genes (*OR*s) are responsible for encoding these olfactory receptor proteins [4]. *OR*s comprise the largest gene family in mammals and are broadly classified into two categories—class-2 and non-class-2—based on their nucleotide sequences [5,6]. In certain studies, non-class-2 *OR*s are referred to as ‘class-1 *OR*s’. Each *OR* encodes a specific olfactory receptor protein that interacts with particular ligands, enabling the discrimination between different odors [7,8].

The observed variation in mammalian olfaction is recognized as a result of anatomical and genomic factors, as described by the aforementioned mechanism. Anatomical features, such as the cribriform plate dimensions [9], generally align with olfactory abilities, exhibiting interspecies variations. Furthermore, an augmented number of *OR* copies within a species denotes an elevated discriminatory capacity and enhanced olfactory significance [10,11]. Primate olfactory organs are comparatively smaller than other mammals, indicating a reduced olfactory capacity within the human species (*Homo sapiens*) [12,13]. Consistently, humans possess approximately 400 copies of *OR*s [14], which is remarkably fewer than the approximate 1000 copies found in mice (*Mus musculus*) or rats (*Rattus norvegicus*) [15]. Both morphological investigations and genomic studies support the diminished importance of olfaction in humans.

The main olfactory organ in mammals is positioned within the respiratory passage, enabling the detection of odors with each inhalation. At this juncture, a query arises: can this sensory system suffice for fully aquatic mammals? Cetaceans, having transitioned into an aquatic environment over 50 million years ago [16,17,18,19], encompass two distinct monophyletic lineages known as baleen whales (Mysticeti) and toothed whales (Odontoceti) [20]. They breathe air solely when they ascend to the water’s surface. Although the frequency of breaths may vary based on activity levels, consistent breathing patterns have been observed across numerous species. For instance, small-toothed whales generally breathe every 1–2 min, killer whales (*Orcinus orca*) breathe no more than every 8 min, and deep-diving species such as sperm whales (*Physeter macrocephalus*) and beaked whales (Ziphiidae) can remain submerged for approximately 1 h [21]. In the case of baleen whales, blue whales (*Balaenoptera musculus*) typically exhibit breathing intervals of approximately 4 min [21]. Humpback whales (*Megaptera novaeangliae*) demonstrate an average interdive breathing interval of 6 min and 45 s during the breeding season. In some instances, particularly among singers (male whales which repeatedly emit patterned sequences of sounds), this interval can extend to approximately 13 min [22,23]. Consequently, cetaceans experience periods of interrupted respiration during dives, leading to the intermittent reception of sensory information through the olfactory modality.

Recently, baleen whales’ olfactory capabilities have been investigated through morphological and genomic studies [24,25,26,27,28,29,30]. The skeletal components of the main olfactory organ, such as the cribriform plate and turbinals, have been observed in common minke whales (*Balaenoptera acutorostrata*) [24,25]. In this species, the nasal mucosa covering the cribriform plate demonstrates similarities to the olfactory mucosa found in terrestrial mammals, as it is lined with pseudostratified columnar epithelium and glandular organs like Bowman’s gland within the lamina propria [26]. Gross and microscopic examinations have supported the presence of the main olfactory organ in bowhead whales (*Balaena mysticetus*) [27,28], and subsequent immunohistochemical staining has identified olfactory nerves in the nasal mucosa of this species [29]. Han et al. [30] conducted a search for *OR*s in seven baleen whale species using publicly available whole genomes, annotating between 54 and 95 intact *OR* copies. The number of *OR*s identified in baleen whales is lower than in other mammals and corresponds to the reduced anatomical complexity of their main olfactory organ.

Both morphological and genomic investigations postulate cetacean hyposmia. This diminished olfactory capability is discernible following the divergence of Artiodactyla and Cetacea [31]. However, baleen and toothed whales exhibit this reduction in distinct manners. While the aforementioned genomic studies suggest a relatively less efficient sense of smell in baleen whales than in terrestrial mammals, they possess a larger repertoire of *OR*s than toothed whales [30,31,32,33]. Analysis of the *olfactory marker protein* gene (*OMP*), which exhibits high expression in the olfactory epithelium and plays a crucial role in olfaction [34,35], indicates that the sense of smell in baleen whales is subjected to purifying selection pressures, whereas toothed whales experience more relaxed selective pressures [36,37]. Furthermore, baleen whales exhibit anatomical features essential for olfaction similar to those found in terrestrial mammals. By contrast, the nasal morphology of extant toothed whales has undergone significant modifications for biosonar signal generation, and it is widely accepted that olfactory structures are absent in this lineage [38,39,40]. Airborne odorants have been proposed to serve as a locating cue for krill, attracting baleen whales through olfactory modality rather than toothed whales [27,31]. Behavioral experiments targeting humpback whales, long-finned pilot whales (*Globicephala melas*), and bottlenose dolphins (*Tursiops truncatus*) have supported this hypothesis [41,42]. The sense of olfaction provides a captivating illustration of how cetaceans interact with their aquatic environment, primarily due to the accelerated evolutionary rate observed in placental mammals’ *OR*s [33]. The remarkable diversification of *OR*s highlights their vital role in shaping species diversity through olfactory perception.

While exploring *OR*s provides a powerful methodology for evaluating olfactory capabilities, it has certain limitations in comprehending the sense of smell [43]. It is important to note that not all *OR*s are exclusively expressed within the olfactory mucosa, which is intricately linked to olfactory reception [44]. The existence of ectopic *OR*s, expressed in various non-olfactory tissues, has been documented [45]. Notably, specific *OR*s in humans and mice exhibit expression in the testis and participate in sperm chemotaxis [46,47,48,49]. Furthermore, a gene known as *OR51E2*, classified as a non-class-2 *OR* is present in nearly all mammalian species, including both baleen and toothed whales [30], and has been identified in the prostate [50]. Hence, the mere presence of *OR*s does not unequivocally signify odor detection capabilities.

Prior investigations have established that baleen whales possess the essential anatomical structures and genetic elements for olfaction; however, these findings alone do not guarantee the existence of a sense of smell based on the same mechanism as observed in other mammals. Therefore, the objective of the present study was to determine whether intact *OR*s are exclusively expressed in the mucosa of the putative main olfactory organ in baleen whales or not. To address this objective, we used the common minke whale as our research subject and extracted total RNA from the nasal mucosa to examine localized *OR* expression.

## 2. Results

### 2.1. Gross Observation

**Figure 1:**
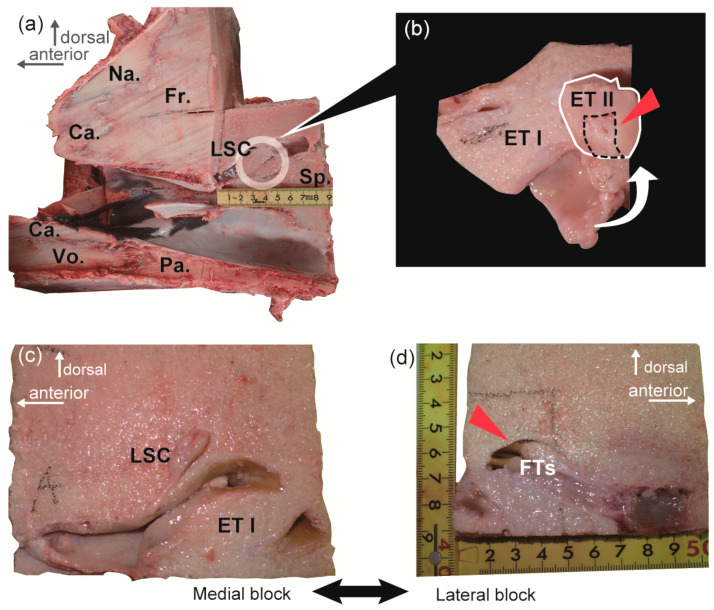
Portions where samples R-001, H-001, and R-006 were harvested. (**a**) A lateral view of a parasagittal section of the cranium from 18NPCK-M001 illustrates the localization of the nasal turbinals (marked by a white circle) within the left nasal chamber. (**b**) An enlarged view of the ethmoturbinals I and II positioned anteriorly to the cribriform plate. The highlighted region, delineated by a white line, represents the posterior end of the ethmoturbinal II. To expose its medial side, this segment was folded over (white arrow). The red arrowhead denotes the site of mucosal sample R-001 extraction (enclosed by a dashed line). Additionally, histological sample H-001 was obtained in proximity to this area. (**c**) A parasagittal section of the left nasal chamber from 18NPCK-M006. This section, depicted in a medial block, provides a lateral view. (**d**) The corresponding lateral side (in medial view) of the section shown in (**c**). This photograph displays two frontoturbinals. Mucosal sample R-006 was collected from the posterior-most region of the dorsal frontoturbinal (indicated by the red arrowhead). Abbr: Ca., cartilage; ET I and II, ethmoturbinal I and II, respectively; Fr., frontal bone; FT, frontoturbinal; LSC, lamina semicircularis; Na., nasal bone; Pa., palatine bone; Sp., presphenoid bone; Vo., vomer bone.

**Figure 2:**
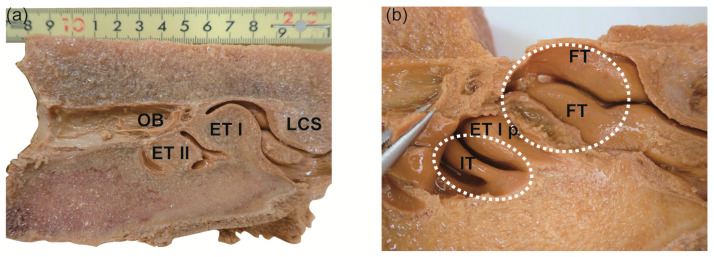
The parasagittal section of the left nasal chamber of 16NPCK-M009. (**a**) Medial view of the nasal chamber and the posteriorly adjoining olfactory bulb chamber. (**b**) A closer medial view of the nasal chamber following removal of ethmoturbinals I and II. The lateral region of the nasal chamber becomes visible. The regions previously occupied by the ethmoturbinals I and II are indicated by circles outlined with white dashed lines. The left and bottom sides correspond to the posterior and ventral directions, respectively. Abbr: ET I and II, ethmoturbinal I and II, respectively; ET I p, ethmoturbinal I posterior part; FT, frontoturbinal; IT, interturbinal; LSC, lamina semicircularis; OB, olfactory bulb.

Three distinct nasal turbinals were observed in the medial aspect of the nasal chamber in all the investigated animals (Figure 1 and Figure 2, Table 1). These structures were identified as the lamina semicircularis, ethmoturbinals I and II. The configuration of these nasal turbinals closely resembles the ‘ethmoturbinates/olfactory recess’ described in common minke whales (Figures 6 and 7 in Godfrey et al. [24]), as well as bowhead whales (Figure 7 in Farnkopf et al. [29]).

The cranial bony block from 16NPCK-M009 exhibited a complicated morphology of nasal turbinals situated laterally to the ethmoturbinals I and II (Figure 2). The ethmoturbinal I was positioned anteriorly to the olfactory bulb chamber and was accompanied ventrolaterally by the posterior part of ethmoturbinal I (Figure 2b, ET I p). Lateral to the ethmoturbinal I, the dorsal region of the nasal chamber was occupied by two slender frontoturbinals (Figure 2b, FT) that corresponded to the same area from which R-006 was obtained (Figure 1c,d). A relatively small turbinal structure, known as the interturbinal (Figure 2b, IT) was located lateral to the ethmoturbinal II.

### 2.2. Expression of the Olfactory Receptor Genes

**Figure 3:**
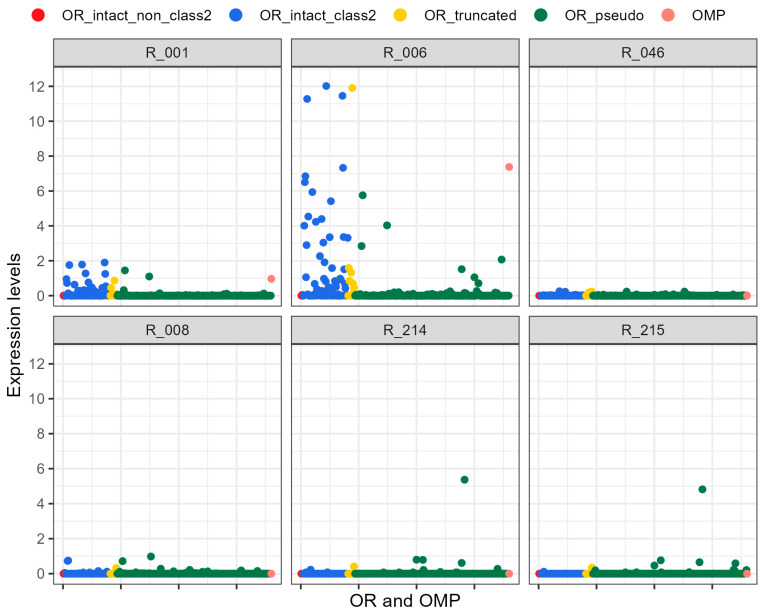
Expression of *OR*s and *OMP*. R-001, R-006, and R-046 correspond to the nasal mucosae of the ethmoturbinal II, frontoturbinal, and anterior nasal chambers, respectively. R-008 originated from the olfactory bulb, and both R-214 and R-215 were obtained from the external skin. The average expressions of intact *OR*s exhibiting non-zero expression were as follows: R-001, 0.370 ± 0.503; R-006, 2.286 ± 3.054; R-046, 0.045 ± 0.068; R-008, 0.146 ± 0.236; R-214, 0.106 ± 0.061; R-215, 0.078 ± 0.038.

Transcriptome sequencing using RNA-seq was performed on the nasal mucosae of the putative olfactory organ (R-001, R-006, R-046), the olfactory bulb (R-008), and the external skin (R-214 and R-215) of common minke whales. The FPKM values of *β-actin* were as follows: R-001, 983; R-006, 285; R-046, 549; R-008, 371; R-214, 161; R-215, 143 (Table 2), indicating successful RNA extraction from all the samples. In this study, 81 intact *OR*s, 12 pseudogenes, and 266 truncated genes were annotated (Supplementary file S1). The maximum expression level (as a percentage of FPKM for *β-actin*) of intact *OR*s in negative controls (the olfactory bulb, R-008, and external skin, R-214 and R-215) was 0.749 of R-008 (Figure 3). The average expression of intact *OR*s with non-zero expression across all samples was 1.014. This expression level was used as the criterion for expression in this study.

**Figure 4:**
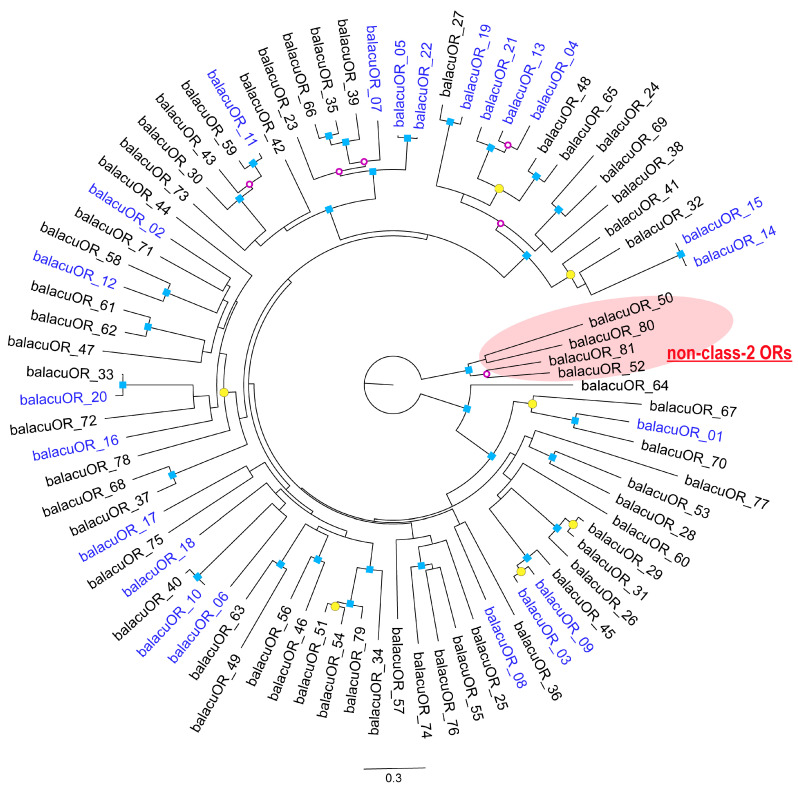
Phylogenetic tree constructed from intact *OR*s. Genes labeled in blue were determined to be expressed in R-006. The root of the tree was established based on non-class-2 *OR*s. Nodes displaying bootstrap values of 100, 86–99, and 71–83 are denoted by blue squares, yellow circles, and open circles outlined in purple, respectively. Nodes with bootstrap values below 70 are not marked.

Among the nasal mucosa samples, R-006 from the frontoturbinal (Figure 1b) exhibited the expression of 22 intact *OR*s, while five of them were also expressed in R-001 from the ethmoturbinal II (Figure 1d). *OMP*, on the other hand was exclusively expressed in R-006, with an expression level greater than 1.014. By contrast, R-046, obtained from the anterior portion of the nasal chamber, did not demonstrate sufficient expression of *OR*s or *OMP* for our criterion of 1.014. Out of the 81 annotated intact *OR*s, all the genes expressed in the nasal mucosa samples belonged to class-2 *OR*s. Although four copies of intact non-class-2 *OR*s were annotated, none were expressed in the six investigated samples (expression levels were between 0 and 0.045). The expressed intact *OR*s did not form a cluster in the phylogenetic tree (Figure 4). Note, that some pseudogenes were highly expressed in the nasal mucosa, and one pseudogene was exclusively expressed in the external skin, as per our investigation.

### 2.3. Histological Staining

**Figure 5:**
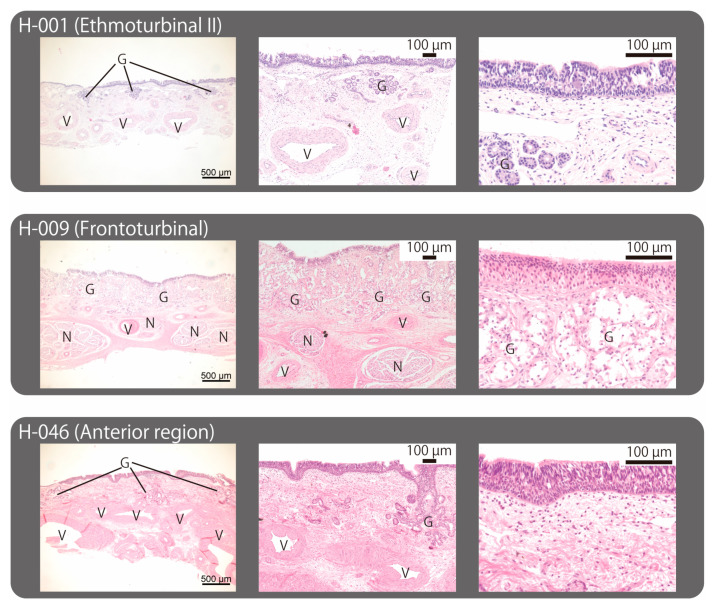
Microscopic views of mucosal samples. The scale bars in the photographs in the far-left, center, and far-right columns are 500 µm, 100 µm, and 100 µm, respectively. The nasal cavity is located at the top of each image. Abbr: G, glands; N, peripheral nerve; V, blood vessel.

Histological examination of the nasal mucosae (H-001, H-009, and H-046) revealed that they were all covered with pseudostratified columnar epithelium and contained glands with orifices (Figure 5, Table 3). These glands appeared to be serous glands, and goblet cells were not observed. In all three mucosae, a nucleus-free zone was observed between the apical surface of the epithelium and the nuclei of supporting cells. This phenomenon was particularly evident in H-009, the sample harvested from the frontoturbinal. Additionally, peripheral nerves were distributed beneath the lamina propria in H-009 (Figure 5).

## 3. Discussion

This study confirmed the specific expression of 22 *OR* copies in the posterior region of the nasal chamber in common minke whales. The *OR*s were predominantly expressed in the posterior portion of the nasal chamber, facing toward the olfactory bulb, with higher expression levels observed in the frontoturbinal region (R-006) and moderately in the ethmoturbinal II (R-001). Previous reports noted that these nasal turbinals are covered with the olfactory epithelium in typical mammals [51]. Furthermore, the *OMP* expression level was highest in sample R-006, indicating that the posterior portion of the frontoturbinal region can be identified as an olfactory region. Although the *OMP* expression in R-001 (0.966) did not meet the criterion of 1.014, it was higher than expression levels observed in R-046, R-008, R-214, and R-215, which showed no *OMP* expression. Moreover, the expression of *OR*s in R-001 was distinctly higher than in R-046, R-008, R-214, and R-215, but lower than R-006 (Table 2). These findings led us to hypothesize that the sampled region R-001, specifically the posterior medial surface of ethmoturbinal II (Figure 1b), encompasses both respiratory and olfactory areas. It is commonly reported that respiratory and olfactory mucosae are distributed in a mosaic-like pattern, and this distribution may hold for cetaceans as well.

In this study, we conducted a microscopic examination of the ethmoturbinal II (H-001), the frontoturbinal (H-009), and the anterior region of the nasal chamber (H-046). These samples were assessed based on histological criteria proposed by Farnkopf et al. [29] and were likely identified as olfactory mucosa. However, it was the proximal region of the frontoturbinal (R-006) that suggested being the olfactory epithelium based on RNA-seq data (Table 3). Notably, the same region (H-009) exhibited a rich abundance of peripheral nerves, indicating its high sensitivity. On the other hand, within H-046, which was obtained from a more anterior region of the nasal cavity, dense clusters of vessels with thick walls were observed (Figure 5). This region may serve as a respiratory area where the vascular epithelium plays a role in thermoregulation. 

Although the expressed *OR*s did not form a distinct cluster in the phylogenetic tree (Figure 4), it should be noted that the present study does not exclude the possibility of a concealed cluster containing *OR*s expressed in unexplored regions. One reason for this is that the distribution of olfactory receptor proteins, which mediate odoriferous stimuli to the olfactory bulb is not uniform across the olfactory epithelium [52,53,54]. Furthermore, the distribution pattern of the olfactory mucosa within the nasal chamber varies among lineages [55,56,57,58]. It is plausible that common minke whales possess additional *OR*s that contribute to their olfactory modality. Identifying such receptors would enhance our understanding of the molecular mechanisms underlying cetacean olfaction.

To comprehend the expression pattern of *OR*s, a thorough anatomical examination of the cetacean nasal chamber is indispensable. Our observations unveiled additional nasal turbinals positioned laterally to ethmoturbinals I and II (Figure 2b, FT and IT), which have been scarcely documented in cetaceans. However, due to the dearth of comprehensive anatomical data encompassing the entire nasal chamber, precise determination of the exact locations from which H-046 and R-046 were obtained remains elusive. Nasal turbinals can be broadly categorized into olfactory and respiratory turbinals, both of which play a pivotal role in unraveling mammalian aquatic adaptation [51,59,60]. In the nasal chamber of common minke whales, the anterior segment from which H-046 and R-046 were harvested is inferred to represent the respiratory region. It is conceivable that baleen whales also possess respiratory turbinals and investigating this structure is warranted in future studies. Although the present study primarily focused on the posterior region adjacent to the cribriform plate, gaining a comprehensive understanding of the entire labyrinthine architecture is crucial [61].

Fundamental anatomical data can function as an atlas during the dissection process. As highlighted by Farnkopf et al. [29], extracting the nasal chamber from the cranial bones of large whales presents a formidable challenge due to their remarkable dimensions, thickness, and robust structure. Identifying the nasal turbinals from sectional images can prove challenging, as their appearance exhibits variations with slight deviations in cutting angles. Moreover, the intricate nature of these structures impedes the efficient penetration of fixation solutions into the tissues. To surmount these sampling difficulties, a comprehensive description of the entire nasal chamber using CT imaging becomes imperative. Determining olfactory epithelium distribution emerges as the next crucial step. Predicting olfactory epithelium locations may be possible based on the surface coloration of the nasal mucosae. While the majority of the nasal mucosa in common minke whales displayed a pale pink hue, R-006, which exhibited distinct olfactory characteristics was obtained from an epithelium displaying a yellowish appearance. However, this coloration may become indistinguishable once the sample is processed in formalin. A previous study tentatively proposed the limited usefulness of pigmentation in describing the distribution of olfactory epithelium in bowhead whales [29], indicating the need for further investigation.

The present study identified a pseudogene exclusively expressed in samples from external skin (Figure 4). This result is considered a form of biologically irregular expression, as the experiment was conducted successfully. The gene, identified as XM_028166200 in mBalAcu1.1 (GenBank accession GCF_949987535.1) is a pseudogene located outside *OR* clusters on the chromosome. Proper *OR* expression is governed by enhancer elements that target genes within a cluster; therefore, *OR*s outside the clusters are likely to fail in normal transcription [62]. The pseudo-*OR* expressed in skin samples is inferred to be one of these *OR*s and might be undergoing pseudogenization.

The entirety of the expressed *OR*s identified in this study exclusively belong to class-2 (Figure 4). Although there remains significant room for exploration, the probability of non-class-2 *OR* expression in common minke whales was presumed to be minimal even when thoroughly screening the entire lining mucosa of the nasal chamber. This presumption is rooted in observations during our dissection, which suggested the absence of the dorsal domain of the olfactory bulb in common minke whales. Non-class-2 olfactory receptors typically transmit input to the dorsal domain of the olfactory bulb. Consequently, we anticipated that non-class-2 *OR*s would not be expressed in the nasal mucosa of an animal lacking this specific region of the olfactory bulb. It has been documented that bowhead whales have also lost the dorsal domain of the olfactory bulb [27,28], and that common minke whales exhibit a dorsoventrally flattened olfactory bulb, akin to bowhead whales.

This study suggests that non-class-2 *OR*s do not partake in baleen whales’ olfactory reception. In mice, non-class-2 *OR*s receive stimuli that trigger avoidance behaviors and project them into the dorsal domain of the olfactory bulb [63]. Hence, our findings imply the loss of the typical avoidance response to specific odorants, such as predators or putrefying substances, in whales. 

Considering that sirenians, another lineage of fully aquatic mammals, still retain their olfactory organs and possess a large repertoire of *OR*s [30,33,64,65], the diminished olfactory ability of baleen whales cannot be solely attributed to their aquatic lifestyle, which restricts continuous respiration. One possible explanation for this lies in the necessity for discerning ingested foods. Anatomically, the esophagus of cetaceans is separated from the airway [66], preventing them from detecting smells emanating from the oral cavity. Genomic research has revealed a degeneration of taste, the other form of chemoreception, in cetaceans [32,67,68]. Furthermore, there is no anatomical description of taste buds in the tongues of baleen whales [69,70,71,72]. Consequently, baleen whales do not rely on chemosensory modalities to evaluate food in their mouths, which may contribute to their reduced olfactory capabilities.

Our genomic and histological investigations suggest that conserved class-2 *OR*s are responsible for olfaction in baleen whales. Specifically, the present study indicates that baleen whales can detect desirable odors, such as those associated with prey and potential mating partners. For instance, sporadically distributed dimethyl sulfide plays a crucial role in olfactory foraging for seabirds in marine environments [73,74,75]. A similar mechanism may operate in baleen whales [27,31]. This foraging strategy poses the risk of the inadvertent ingestion of marine plastic debris [76], underscoring the importance of assessing olfactory acuity in baleen whales. The present study establishes a foundational connection between rorqual anatomy and genome. Subsequent inquiries hold promise for elucidating the natural, social, and behavioral biology of these creatures, contributing to their conservation efforts.

## 4. Materials and Methods

### 4.1. Sample Collection

The present study examined common minke whales obtained from the coastal regions of Japan. The specimens were acquired from seven animals (Table 1). In 2016, 16NPCK-M009 and 16NPCK-M012 were procured during the second phase of the Japanese Whale Research Program under a special permit in the Western North Pacific (JARPNII). In 2018, 18NPCK-M001, 18NPCK-M006, and 18NPCK-M008 were obtained during the New Scientific Whale Research Program in the Western North Pacific (NEWREP-NP). In 2019, 19SK214 and 19SK215 were acquired during Japanese commercial whaling operations. The research programs adhered to the regulations outlined in Article VIII of the International Convention for the Regulation of Whaling (ICRW). All samples were collected in accordance with legal procedures.

The whales were harvested in the offshore waters of Hokkaido, Japan, and transported to fishing facilities for processing. To obtain the nasal mucosa, we meticulously dissected the occipital bones of common minke whales and extracted their brains to identify the entrances to the left and right olfactory tract tunnels. The head was then trimmed using a chain saw to create a bony block that encompassed the olfactory bulb tract and the nasal chamber. Upon observing the medial view of the sections (Figure 1a and Figure 2a), we noted three prominent nasal turbinals, namely the lamina semicircularis and the ethmoturbinals I and II, arranged from the dorsal to ventral position [77], providing important indications for orientation.

The bony block, measuring 12 cm anteroposteriorly, 7 cm dorsoventrally, and 5 cm transversally was obtained from the left side of 16NPCK-M009. Subsequently, it was fixed in 10% formalin at the collection site and used for gross examination of the nasal chamber. A 5 mm square piece of mucosa was extracted from the posterior end of the frontoturbinal from the block, which was labeled H-009 for histological analysis.

We obtained two mucosal samples from 18NPCK-M001. Both samples were collected from the left side nasal mucosa of ethmoturbinal II, situated in front of the cribriform plate (Figure 1a,b). One sample was designated R-001 and frozen in RNA-later (Thermo Fisher Scientific Inc., Waltham, MA, USA) for subsequent RNA-seq analysis. The other sample, H-001 was fixed in 10% formalin for microscopic examination.

Another mucosal sample was acquired for RNA-seq analysis from 18NPCK-M006. The nasal chamber on the left side was carefully trimmed off the head (Figure 1c,d), promptly frozen, and transported to the laboratory. Subsequently, a 5 mm square mucosal sample was excised from the posterior end of the frontoturbinal region and designated R-006. 

The bony block derived from specimen 18NPCO-M046 was sectioned in a transverse manner yielding two mucosal pieces extracted from the anterior portion of the right nasal chamber. The mucosal piece intended for RNA-seq analysis was labeled R-046, enclosed in a vinyl bag containing RNA-later, and stored in a freezer for preservation. Additionally, an adjacent mucosal piece, designated H-046 was collected and preserved in 10% formalin for subsequent microscopic examination. 

To facilitate comparisons of gene expression across different organs, samples were also obtained from the olfactory bulb and external skin. We dissected 18NPCK-M008, excised the anterior 5 mm tip of the right olfactory bulb, and stored it in a freezer with RNA-later. This particular piece was labeled R-008. Furthermore, external skin samples were obtained from specimens 19SK214 and 19SK215, identified as R-214 and R-215, respectively, and preserved in a freezer.

The sampling procedures were recorded through both handwritten notes and digital macrophotography (Tough TG-5; Olympus Corporation, Tokyo, Japan). We quantified the postmortem interval as the duration between the animal’s capture by boats above the sea and the preservation of the collected sample in either a freezer or formalin. The maximum recorded postmortem interval was 8.5 h (Table 1).

### 4.2. Gross Observation

The bony block obtained from 16NPCK-M009 was bisected along the medial wall to reveal the inner side of the nasal chamber. The cross-sections of the lamina semicircularis and ethmoturbinals I and II were observed, as shown in Figure 2. Upon removing the skeletal tissues of the medial nasal turbinals (ethmoturbinals I and II), the laterally positioned turbinals became visible. The anatomical nomenclature used to identify the nasal turbinals was based on Ito et al. [78] and supplemented by relevant references [77,79].

### 4.3. RNA Expression

The RNA expression analysis in the present study employed the same methods described by Kishida et al. [44]. The *OR* genes were queried against the common minke whale genome assembly (GenBank accession GCA_000493695.1) [80] using the TBLASTN program in the BLAST+ v. 2.6.0 package [81] with a cut-off E-value of 1 × 10^−5^. Deduced amino acid sequences of all intact *OR*s from green anole (*Anolis carolinensis*) and western clawed frog (*Xenopus tropicalis*), cow (*Bos tauros*), and mouse identified by Niimura [6] and Niimura et al. [82] were used as queries. Each obtained sequence was searched against the GenBank protein database using the BLASTX program [81]. If its best hit did not correspond to an *OR*, it was discarded. A sequence was deemed a non-functional pseudogene if it contained premature stop codons and/or frameshifts, or if it lacked five or more consecutive amino acids, including a transmembrane domain. Sequences interrupted by contig-gaps, though not classified as pseudogenes were labeled ‘truncated’. 

FATE (https://github.com/Hikoyu/FATE, accessed on 16 January 2023) was employed to search the common minke whale genome assembly (GenBank accession GCA_000493695.1) for *OMP*, using the annotated query sequence NW_006728793.1, identified as *OMP*, from GenBank Refseq GCF_000493695.1. The resulting single sequence found in GCA_000493695.1 was used for *OMP* mapping.

Total RNA was extracted from the nasal chamber mucosae (R-001, R-006, and R-046), olfactory bulb (R-008), and external skin (R-214, R-215) using the RNeasy Mini Kit (Qiagen, Hilden, Germany) following the manufacturer’s guidelines. The olfactory bulb and external skin samples served as negative controls. The extracted RNA was used to construct paired-end sequencing libraries using the TruSeq Stranded mRNA LT Sample Prep Kit (Illumina Inc., San Diego, CA, USA). Subsequently, an Illumina NovaSeq platform (2 × 101 bp) was employed for sequencing, generating RNA-seq reads of the following sizes: R-001, 5.89 G bp; R-006, 4.57 G bp; R-046, 5.54 G bp; R-008, 5.37 G bp; R-214, 5.35 G bp; R-215, 6.03 G bp. Low-quality sequences and adapters were removed using Trimmomatic [83] v. 0.38 with the following parameters: ILLUMINACLIP: TruSeq3-PE-2.fa: 2:30:10, LEADING: 20, TRAILING: 20, SLIDINGWINDOW: 4:20, and MINLEN: 36. HISAT2 [84] v. 2.1.0 with default parameters was used to map trimmed RNA-seq reads to the conspecific genome assembly. Gene expression levels were quantified using fragments per kilobase of exon per million mapped fragments (FPKM) values with Cufflinks [85,86] v. 2.2.1 after removing duplicate reads. *OR* expression level was calculated by dividing it by *β-actin* gene and multiplying it by 100 to provide an expression percentage. Data analyses were performed using R (https://www.R-project.org, accessed on 28 July 2023) v. 4.2.2, with plots generated using the tidyverse package [87] v. 1.3.2 and ggplot2 [88] v. 3.4.0. 

The annotated intact *OR*s of common minke whales were incorporated into a phylogenetic tree. The nucleotide sequences were aligned using MAFFT [89,90] v. 7. A suitable model was determined by ModelTest-NG [91,92] v. 0.1.7. Subsequently, a GTR + I + G4 model was selected, and the phylogenetic tree was constructed using RAxML-ng [93] v. 1.2.1 with the root set as non-class-2 *OR*s. The annotated intact *OR*s were numbered in descending order by FPKM values.

### 4.4. Histology Staining

The mucosal specimens underwent standard histological techniques. The samples were dehydrated using a series of ethanol concentrations and then cleared with xylene. Following infiltration and embedding in paraffin wax (melting point 56–58 °C), they were sectioned using a rotary microtome (PR-50; Yamato Kohki Industrial Co., Ltd., Saitama, Japan) into slices measuring 4–6 μm. These sections were spread out on warm water, carefully transferred onto glass slides, and dried in an incubator at 60 °C for 30 min. During the staining process, they were immersed in deparaffinization solution, hydration medium, and stain solution. Following mounting, the epithelial samples were examined and photographed using a digital microscope (VHX-7000; Keyence, Osaka, Japan; Axioscope 5 and Axiocam 503 color; Carl Zeiss, Jena, Germany). We evaluated whether these epithelial tissues qualified as olfactory epithelium based on criteria proposed by Farnkopf et al. [29]: epithelium constructed of basal cells, supporting cells, and olfactory sensory neurons; the presence of Bowman’s glands; the absence of goblet cells, and the distance between the apical surface and nuclei of the supporting cells.

## Figures and Tables

**Table 1:** List of specimens.

Location	Year	ID	Sex	Body Length (m)	PMI ^†^ (h)	Description of Specimen	Side	Techniques Used	Sample ID
Kushiro, Hokkaido	2016	16NPCK-M009	M	7.01	4.5	Nasal mucosa	L	Histology	H-009
Kushiro, Hokkaido	2018	18NPCK-M001	M	5.50	6	Nasal mucosa (Ethmoturbinal II)	L	RNA-seq Histology	R-001 H-001
		18NPCK-M006	M	7.09	6	Nasal mucosa (Frontoturbinal)	L	RNA-seq	R-006
		18NPCK-M008	F	4.62	6	Olfactory bulb	R	RNA-seq	R-008
Abashiri, Hokkaido	2018	18NPCO-M046	F	7.30	5	Nasal mucosa (Anterior portion)	R	RNA-seq Histology	R-046 H-046
Kushiro, Hokkaido	2019	19SK214	M	6.96	8.5	External skin	-	RNA-seq	R-214
		19SK215	M	7.33	5.5	External skin	-	RNA-seq	R-215

^†^ PMI means the post-mortem interval.

**Table 2:** FPKM and calculated expression percentage.

	R-001	R-006	R-046	R-008	R-214	R-215
*β actin* (FPKM)	983	285	549	371	161	143
*OMP*						
FPKM	9.510	21.101	0	0	0	0
Expression ^†^	0.966	7.378	0	0	0	0
Average of expressing intact *OR*s						
FPKM	3.640 ± 4.946	6.537 ± 8.735	0.244 ± 0.372	0.543 ± 0.876	0.171 ± 0.098	0.112 ± 0.054
Expression ^†^	0.370 ± 0.503	2.286 ± 3.054	0.045 ± 0.068	0.146 ± 0.236	0.106 ± 0.061	0.078 ± 0.038

^†^ Expression (%) was calculated as follows: FPKM of a gene was divided by that of β actin, then multiplied by 100.

**Table 3:** Results of RNA-seq and histological observation of mucosal samples.

Description of Specimen	Sample ID	*OR* Expression	*OMP* Expression	Pseudostratified Columnar Epithelium	Bowman’s Glands	Absence of Goblet Cells	Nucleus Free Zone	Peripheral Nerve
Ethmoturbinal II	H-001 R-001	low	low	Y	Y	Y	Y	N
Frontoturbinal	H-009 R-006	Y	Y	Y	Y	Y	Y	Y
Anterior portion	H-046 R-046	N	N	Y	Y	Y	Y	N

## Data Availability

All sequence reads were deposited in the DDBJ Sequence Read Archive under BioProject accession no. PRJDB16252.

## Supplementary Materials

The following supporting information can be downloaded at: https://www.mdpi.com/article/10.3390/ijms25073855/s1.
